# Discrimination and Perceived Cultural Mismatch Increase Status-Based Identity Uncertainty

**DOI:** 10.1177/01461672231163736

**Published:** 2023-04-07

**Authors:** Sierra H. Feasel, Tessa L. Dover, Payton A. Small, Brenda Major

**Affiliations:** 1 University of California, Santa Barbara, USA; 2 Portland State University, OR, USA; 3 Vassar College, Poughkeepsie, NY, USA

**Keywords:** status uncertainty, social mobility, discrimination, cultural mismatch

## Abstract

Periods of social mobility, such as attending college, can challenge one’s status-based identity, leading to uncertainty around one’s status in society. Status uncertainty is associated with poorer well-being and academic outcomes. Little is known, however, about what experiences lead to status uncertainty. The current longitudinal study investigated discrimination experiences and cultural mismatch as predictors of status uncertainty. We propose that discrimination indirectly predicts increased status uncertainty by increasing perceived cultural mismatch with the university. Participants were Latinx college students, all of whom were low-income and/or first generation to college. Discrimination experiences were measured at the end of participants’ first year. Cultural mismatch and status uncertainty were measured at the end of Year 2. Status uncertainty was measured again at the end of Year 3. Results indicated that students who experienced more frequent discrimination felt more cultural mismatch 1 year later, and, in turn, reported increased status uncertainty over the following year.

## Introduction

Many students from low socioeconomic status (SES) backgrounds see graduating from college as the key to opening new doors for the future. Indeed, a college degree has many benefits, including increased earnings, better career prospects, and more social opportunities, especially for students coming from less advantaged backgrounds (Abel & Deitz, 2014; Brand & Xie, 2010). However, when these students reach college, the road to graduation is often rocky and paved with unique challenges related to their status. Students from low-SES backgrounds may experience discrimination based on their SES (Van Dyke et al., 2016) and perceive a cultural mismatch between the interdependent norms of their working-class backgrounds and the independent norms of college environments (Stephens, Fryberg, et al., 2012). This path is even harder for low-SES racial and ethnic minority students, who often experience racial discrimination in addition to SES discrimination as they pursue upward mobility (Bird & Bogart, 2001; Ren et al., 1999). Thus, students who come from both low-SES and racial/ethnic minority backgrounds are at especially high risk of facing these aversive experiences during college (Vasquez-Salgado et al., 2015).

Recent research has begun to examine status-based identity, or the subjective experience of an individual’s SES, as a framework to better understand the experience of social mobility (Destin et al., 2017). While related to subjective status, this framework is more capable of capturing the flexibility of the experience of one’s SES, which can change even from moment to moment. Status-based identity can be challenged and threatened during status transition periods, such as attending college (Destin, 2019). Students from lower status backgrounds may find it difficult to reconcile differences between their SES growing up and their changing place in society. For example, many low SES and first-generation college students face a cultural mismatch when entering college environments (Stephens, Fryberg, et al., 2012). These students often endorse interdependent cultural norms, while many universities prioritize independence. This mismatch is associated with lower sense of fit and lower grades throughout college (Phillips et al., 2020). These challenges may be exacerbated among students of color who also face racial discrimination demonstrating that their racial identity is devalued in society. These resulting feelings of uncertainty around where one stands in society, known as status-based identity uncertainty or status uncertainty, have negative implications for academic outcomes and overall well-being (Destin et al., 2017, 2019).

Although status-based identity uncertainty is thought to be most common during periods of social mobility, research has yet to examine the specific experiences that lead to status uncertainty. Given the negative effects of status uncertainty on well-being and academic outcomes, it is important to understand predictors of this experience. The current study longitudinally examines the role of discrimination experiences and cultural mismatch within the university context in predicting changes in status uncertainty during college for low-SES ethnic minority college students. Prior research has shown this population of college students experiences lower levels of belonging in college, poorer well-being, and lower grades compared with White students from higher-SES backgrounds (Azmitia et al., 2018; Phillips et al., 2020; Stephens, Fryberg, et al., 2012). These social class differences highlight the importance of understanding the experiences of these students. The present research contributes to this goal by working toward a more holistic understanding of status uncertainty and experiences that lead to this uncertainty. The current study also investigates how negative experiences related to students’ social status and their race/ethnicity are related to one another.

### Status-Based Identity and Status-Based Identity Uncertainty

Destin and colleagues (2017) proposed the concept of status-based identity as a framework to understand the subjective experience of status. Status-based identity is the subjective meaning and value that people attach to their SES (Destin et al., 2017). Status-based identity can change over a person’s lifetime and can even differ from moment to moment. Traditional measures of subjective SES are not equipped to capture this flexibility. Status-based identity provides a fuller picture by drawing from narrative identity, social identity, and future identity. Narrative identity helps people make sense of their changing place in society (J. M. Adler et al., 2016; Destin & Debrosse, 2017). Social identity explains the desire to form meaningful connections within one’s social context (Tajfel & Turner, 1979). Finally, future identity describes people’s vision of their future self and their future life, which often includes an expectation of one’s future status (Oyserman et al., 2015).

Periods of social mobility can challenge an individual’s status-based identity by challenging these three aspects of identity (Destin, 2019). For example, coming from a different background than other students can cause students to feel like they don’t fit in with their college peers, threatening the student’s social identity as a student at their university. Low-SES students often have a hard time finding a community of other students who share their background (Azmitia et al., 2018) and thus struggle to develop an identity as a college student (Jury et al., 2017). Entering into a college environment that is characterized by more independent norms can also create a disconnect between a student’s home life and their new college life, threatening the student’s narrative identity, or the way they make sense of their life journey. These students can often experience guilt around their educational achievement and the associated opportunities that they had access to that other family members did not (Covarrubias & Fryberg, 2015). Especially for low-SES racial and ethnic minority students, experiencing discrimination may serve as a status threat that makes salient the negative views that others may hold about their ethnic group and/or SES (Orbe, 2004). Knowing that society devalues their social identities may lead students to have lower hopes for their future after college, threatening their future identity.

These different threats to one’s status-based identity can lead to status uncertainty, or uncertainty around one’s standing in society. This is characterized by having conflicting beliefs about one’s social standing or experiencing conflict between one’s past and one’s future in society (Destin et al., 2017). An initial test of the effects of status uncertainty found that higher levels of status uncertainty were associated with lower self-esteem and lower satisfaction with life (Destin et al., 2017). Researchers replicated the negative association between status uncertainty and self-esteem and satisfaction with life among Latina college students (Castillo-Lavergne & Destin, 2019). Destin and colleagues (2019) found that experimentally inducing feelings of status uncertainty decreased college students’ motivation to engage in individual studying, peer opportunities, and seek faculty support. These results were replicated in a longitudinal study that found that status uncertainty predicted reduced academic efficacy, which, in turn, led to poorer academic performance (Destin et al., 2019). Despite these findings, little is known about what predicts status uncertainty. The current research aimed to examine predictors of status uncertainty during a common period of social mobility: college. Examining experiences during college that lead to status uncertainty may uncover ways to reduce the experience of status uncertainty and its negative effects, and will also help to better understand the experience of low-SES and/or first-generation college students from racial/ethnic minority backgrounds.

### The Paradox of Upward Social Mobility

Upward social mobility is associated with a variety of beneficial outcomes, including greater access to resources and opportunities. Typically, upward social mobility is also positively associated with health. Higher SES, measured both objectively and subjectively, reliably predicts better psychological health and well-being, including lower rates of psychiatric disorders, as well as better physical health, including lower mortality rates and decreased risk for disease (N. E. Adler et al., 1994; Braveman et al., 2010; Cohen et al., 2010; Destin et al., 2019; Dohrenwend, 1990; Fujishiro et al., 2010). Surprisingly, however, research on social mobility suggests that despite the many benefits that accrue from higher SES, climbing the social ladder may come at a cost to physical health for racial and ethnic minorities, especially those from low-SES backgrounds (Brody et al., 2013; Destin, 2019; Geronimus et al., 2006). For example, college completion is associated with poorer rather than better physical health for low-SES Black and Hispanic students who attend college (Gaydosh et al., 2018). And health disparities between White and Black Americans are more pronounced at higher rather than lower levels of SES (Williams & Collins, 1995).

We propose that one key factor likely to contribute to feelings of status uncertainty among this population is the experience of discrimination. As they matriculate to college, low-SES racial and ethnic minorities often face subtle as well as overt discrimination based on either their race/ethnicity or SES in their new middle- and upper-class, and typically majority White, college environment (Cole & Omari, 2003; Geronimus et al., 2006; Ren et al., 1999). The pervasiveness of racial discrimination in college and its negative impact on the college experiences of racial/ethnic minority students has been well-documented (Banks, 2010; Hwang & Goto, 2008; O’Hara et al., 2012). Low-SES racial/ethnic minority individuals also face discrimination based on their SES (Cole & Omari, 2003; Ren et al., 1999). Bird and Bogart (2001), for example, found that the majority of racial minorities in their study reported experiencing both race-based and SES-based discrimination in a health care context. While the effects of racial discrimination have been more thoroughly studied, it is important to consider both types of discrimination because they both can have negative effects. Because race is a more visible social identity, racial discrimination may be more common (Fernandez & Benner, 2022). But SES discrimination still independently has been shown to negatively affect various outcomes, including sleep and inflammation (Van Dyke et al., 2016, 2017).

Experiencing discrimination in college based on either one’s race or SES can lead to feelings of lack of fit within the university context. Cultural mismatch theory posits that first-generation and/or low-SES students experience difficulties transitioning to college because of the clash between the interdependent norms of their working-class backgrounds and the independent norms of predominantly middle- and upper-class college environments (Stephens, Fryberg, et al., 2012). For example, at home, these students take on tasks such as providing advocacy for parents, taking care of their siblings, and working extra jobs to help financially support their families (Covarrubias et al., 2019). These types of skills, however, are not ones that are valued in a typical college environment. This can cause students to feel that they don’t belong in college, which, in turn, can negatively impact their academic performance and stress levels (Stephens, Fryberg, et al., 2012; Stephens, Townsend, et al., 2012). Feelings of cultural mismatch may be especially present among low-SES students who are members of racial and ethnic minority groups, who can also experience a clash between norms associated with their racial or ethnic background and the university context (Birani & Lehmann, 2013; Nguyen & Nguyen, 2020). For example, interdependent cultural norms that are associated with a working-class background, such as familial obligation values, are also common in Latino culture (Fuligni, 2001; Vasquez-Salgado et al., 2015). Because of these similarities in cultural values, Latinx students’ experience of cultural mismatch can stem from both their status-based and ethnic identities. Destin et al. (2017) theorized that cultural mismatch triggers status-based identity uncertainty, although this hypothesis has not yet been tested.

Although little research has empirically examined predictors of perceived cultural mismatch, it has been theorized that cultural mismatch results from social identity threat when students’ cultural values are deemphasized in their university context (Hecht et al., 2021). Discrimination threatens students’ social identities as a student from a low-SES background, a first-generation college student, and a member of their racial/ethnic group (Verkuyten et al., 2019). Thus, we hypothesize that more frequent experiences of racial/ethnic or SES discrimination in college will lead students to feel more cultural mismatch within the university context. Greater feelings of cultural mismatch, in turn, will lead to increased status uncertainty by causing students to question their belongingness in college and struggle to connect their background with their new college environment (Destin et al., 2017).

### The Current Study

**Figure 1.**
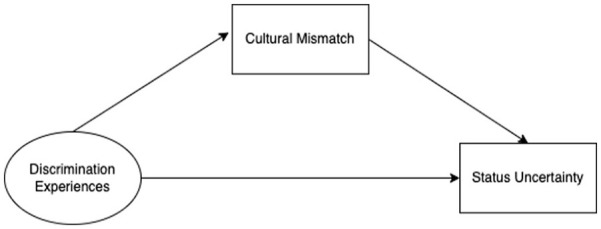
Conceptual Mediation Model.

The current research investigated the role that discrimination experiences and perceived cultural mismatch play in the process leading to status uncertainty among ethnic minority students who either come from low-SES backgrounds or are first-generation college students pursing upward mobility. We proposed that experiencing discrimination in college based on race/ethnicity and/or status indirectly predict increases in status uncertainty by increasing feelings of cultural mismatch with the university (Figure 1). Importantly, we hypothesized that these effects would hold even when controlling for traditional measures of subjective and objective status. This would demonstrate that status uncertainty captures the subjective and flexible experience of status, rather than objective or perceived status at one point in time. We tested this model among a sample of low-income and/or first-generation to college Latinx students. We selected this sample based on the premise that these students are particularly likely to experience both discrimination and feelings of cultural mismatch based on their racial/ethnic identity and their SES (Bird & Bogart, 2001; Nguyen & Nguyen, 2020).

## Method

The current study was not preregistered. Materials and data can be accessed through the Open Science Framework: https://osf.io/25anw/?view_only=da0ab1d15ab14fce98cfe32e2be9d0ef

### Participants

All participants were undergraduates at the University of California, Santa Barbara (UCSB). Several weeks before the start of the Fall 2016 school year, all incoming first year students who self-identified as Latinx, and were identified by the university as either a first-generation student or from a low-income household with family income less than $50,000,^1^ were invited to participate in a study about the transition to college (*N* = 818). Four-hundred and four students indicated interest in participation and provided contact info. All interested students were contacted and the first 300 to respond were invited to participate during the first 3 weeks of Fall quarter (T1). Sample size was determined by the budget available for participant payment.

The inclusion criteria were measured again in the lab by asking participants to indicate their racial or ethnic identity, their annual family income during high school, and their parents’ highest level of education. Students were retained in the sample if they identified as Latinx, and they either had a family income of less than $50,000 or were first-generation college students (with neither of their parents graduating college). Nineteen students did not meet the inclusion criteria, leaving a T1 sample size of 274 low-income/first-generation Latinx incoming college students. Twenty-one participants were missing measures of discrimination experiences and 16 participants were missing the status uncertainty measure. These participants were excluded from the current analyses, leaving a final sample size of 237 participants. A post hoc power analysis using simulations conducted using pwrSEM (Wang & Rhemtulla, 2021) indicated that this sample size gave us 95% power to detect the observed indirect effect of discrimination experiences on status uncertainty through cultural mismatch. Participant ages ranged from 18 to 20 years; the majority were women (70.8%) and first-generation college students (64.4%); self-reported family income ranged from $3,000 to $165,000 (*Mdn* = $35,000, *M* = $41,926, *SD* = $27,046). More than 75% of the sample was from a low-income background. Approval for this study was obtained from the university’s institutional review board (IRB).

### Procedures

#### Time 0

T0 assessment occurred during the first few weeks of the start of participants’ freshman year. Data collection occurred at an on-campus laboratory where informed consent was obtained and measures of demographic information were completed. These data come from a larger longitudinal dataset, and participants completed a number of other measures not relevant to the current study (see Dover et al., 2020). Participants were compensated $40.

#### Time 1

T1 assessment occurred at the end of students’ first year of college. Participants came to the laboratory to complete a measure of the frequency with which they had experienced discrimination at college over the prior year. Participants were compensated $40.

#### Time 2

T2 assessment occurred at the end of students’ second year of school. Participants came to the laboratory to complete measures of status-based identity uncertainty and perceived cultural mismatch. They were compensated $40 for their participation.

#### Time 3

T3 assessment occurred at the end of students’ third year of college. Participants completed the same measure of status-based identity uncertainty from T2 and were compensated $40.

### Primary Measures

#### Objective Social Status

Objective social status was computed as a composite of self-reported family income (*M* = 42,000, *SD* = 27,000, *Mdn* = 35,000, minimum = 3,000, maximum = 165,000), mother’s education (44.4% did not graduate high school), father’s education (49.3% did not graduate high school), and first-generation student status (61.3% first-generation college students) reported at T0. Each student was dummy-coded as 0 or 1 on these four measures (e.g., for self-reported family income, students were coded 1 if they were not from a low-income family and made more than $50,000 and were coded as 0 if they were from a low-income family and made less than or equal to $50,000; for mother’s education, students were coded as 1 if their mother did graduate high school and were coded as 0 if their mother did not graduate high school). These dummy-coded variables were added together to create a composite of objective social status that ranged from 0 to 4 with students who scored a 0 being first-generation students from low-income families whose mother and father did not graduate high school (*M* = 2.19, *SD* = 1.15).

#### Subjective Social Status

Subjective social status was assessed at T0 (*M* = 5.31, *SD* = 1.67), T2 (*M =* 5.11, *SD =* 1.38), and T3 (*M =* 4.96, *SD =* 1.40) using the Scale of Subjective Status (N. E. Adler et al., 2000). Participants viewed a ladder with 10 steps and were instructed:

Think of this ladder as representing where people stand in the United States. At the top of the ladder are people who are the best off—those who have the most money, the most education, and the most respected jobs. At the bottom are the people who are the worst off—who have the least money, least education, and the least respected jobs or no job. The higher up you are on this ladder, the closer you are to the people at the very top; the lower you are, the closer you are to the people at the very bottom. Select the number corresponding to the location you would place yourself at this ladder today.

#### Status-Based Identity Uncertainty

Status uncertainty was measured using the 11-item status-based identity uncertainty scale (Destin et al., 2017). Items assessed the level of uncertainty participants felt around their status-based identity (e.g., “*My beliefs about where I stand in society often conflict with one another”* and *“I spend a lot of time wondering about where I stand in society”*).

Responses ranged from 1 (*Strongly Disagree*) to 7 (*Strongly Agree*), with higher numbers indicating greater status uncertainty. Responses were averaged to compute an index of status-based identity uncertainty at both T2 (α = .87, *M =* 3.73, *SD =* 0.94) and T3 (α = .91, *M =* 3.35, *SD =* 1.12). Status uncertainty significantly decreased from T2 (*M* = 3.73, *SD* = 0.94) to T3 (*M* = 3.35, *SD* = 1.12), *t*(164) = 4.52, *p* < .001. On average, students experienced less status uncertainty at the end of their third year of college (i.e., felt more certain about where they stand in society) compared with the end of their second.

#### Discrimination Experiences

To assess discrimination experiences, we modeled discrimination using a latent variable indicated by measures of racial discrimination and status discrimination. The correlation among these two measures was high at all timepoints: T0 (*r* = .64, *p* < .001), T1 (*r* = .65, *p* < .001), T2 (*r* = .70, *p* < .001), T3 (*r* = .72, *p* < .001).

##### Racial Discrimination

Racial discrimination was measured using a 9-item scale adapted from Williams et al. (1997). Participants responded on a scale from 1 (*Never*) to 5 (*All the time*) how often during the past year they had experienced unfair treatment because of their race/ethnicity (e.g., “*You were treated with less courtesy than other people are*”). Responses were averaged to form a composite at T0 (α = .88, *M =* 2.50, *SD =* 1.00), T1 (α = .92, *M* = 1.87, *SD* = 0.77), T2 (α = .90, *M* = 1.83, *SD* = 0.73), and T3 (α = .92, *M* = 1.83, *SD* = 0.76).

##### Status Discrimination

Status discrimination was measured with a single item: “During the past year at UCSB, how often did you experience discrimination due to your social class/background?” Participants responded on a scale of 1 (*Never*) to 5 (*All the time*). Status discrimination was measured at T0 (*M* = 2.39, *SD* = 1.04), T1 (*M* = 1.68, *SD* = 0.93), T2 (*M* = 1.78, *SD* = 0.97), and T3 (*M* = 1.70, *SD* = 0.91).

#### Cultural Mismatch

Cultural mismatch was measured using a 7-item scale measuring how well they felt their background fit in at college (e.g., “*People at UCSB do not understand my background*” and “*I have to change myself to fit in at UCSB*”). Responses were averaged to create a reliable index of cultural mismatch at T2 (α = .85, *M* = 3.26, *SD* = 1.22) and T3 (α = .82, *M* = 3.34, *SD* = 1.17).

## Results

### Data Analysis Strategy

We hypothesized that experiencing discrimination at college (T1) would lead to increased status uncertainty (T3) via the mediator of greater feelings of cultural mismatch (T2). We used latent variables and a structural equation modeling approach to test this model. We ran the proposed mediational model using *Mplus* version 8.0 (Muthén & Muthén, 1998–2017). T0 objective and subjective status were included as covariates. We also controlled for T2 status uncertainty when predicting T3 status uncertainty to model change over time. See Supplemental Materials for alternative models that were tested.

### Main Results

#### Correlations Among Study Variables

Means, standard deviations, and correlations between observed variables are included in Table 1. As hypothesized, racial/ethnic discrimination, SES discrimination, and cultural mismatch were prospectively associated with status uncertainty at T3. Status uncertainty at T2, *r*(198) = .01, *p =* .943, or T3, *r*(169) = −.06, *p* = .470, was not significantly associated with subjective status at college entry, demonstrating that the experience of status uncertainty is not unique to individuals who perceive themselves to be lower on the social ladder. T0 objective status was significantly correlated with status uncertainty at T3, *r*(175) = −.20, *p* = .007, but not at T2, *r*(206) = −.10, *p* = .152. Students higher in objective SES, measured by family income, parental education, and first-generation status, may be at lower risk of experiencing status uncertainty later in college. Recall, however, that income levels in the current data were skewed, with most students having low objective SES; a larger range may be needed to better understand the correlations between status and status uncertainty.

**Table 1.** Means, SDs, and Correlations Among Study Variables.

Measure	*M* (*SD*)	1.	2.	3.	4.	5.	6.	7.
1. T2 status uncertainty	3.73 (0.94)	—						
2. T3 status uncertainty	3.35 (1.12)	.53***	—					
3. T0 objective SES	2.19 (1.15)	−.10	−.20**	—				
4. T0 subjective SES	5.31 (1.67)	.01	−.06	.12^†^	—			
5. T1 racial discrimination	1.87 (0.77)	.16*	.24**	−.11^†^	−.05	—		
6. T1 SES discrimination	1.68 (0.93)	.06	.19*	−.06	−.13^†^	.65***	—	
7. T2 cultural mismatch	3.26 (1.22)	.31***	.44***	−.14*	−.28***	.51***	.41***	—

*Note.* SES = socioeconomic status.

† *p* < .1. *p < .05. **p < .01. ***p < .001.

#### Model Results

**Figure 2.**
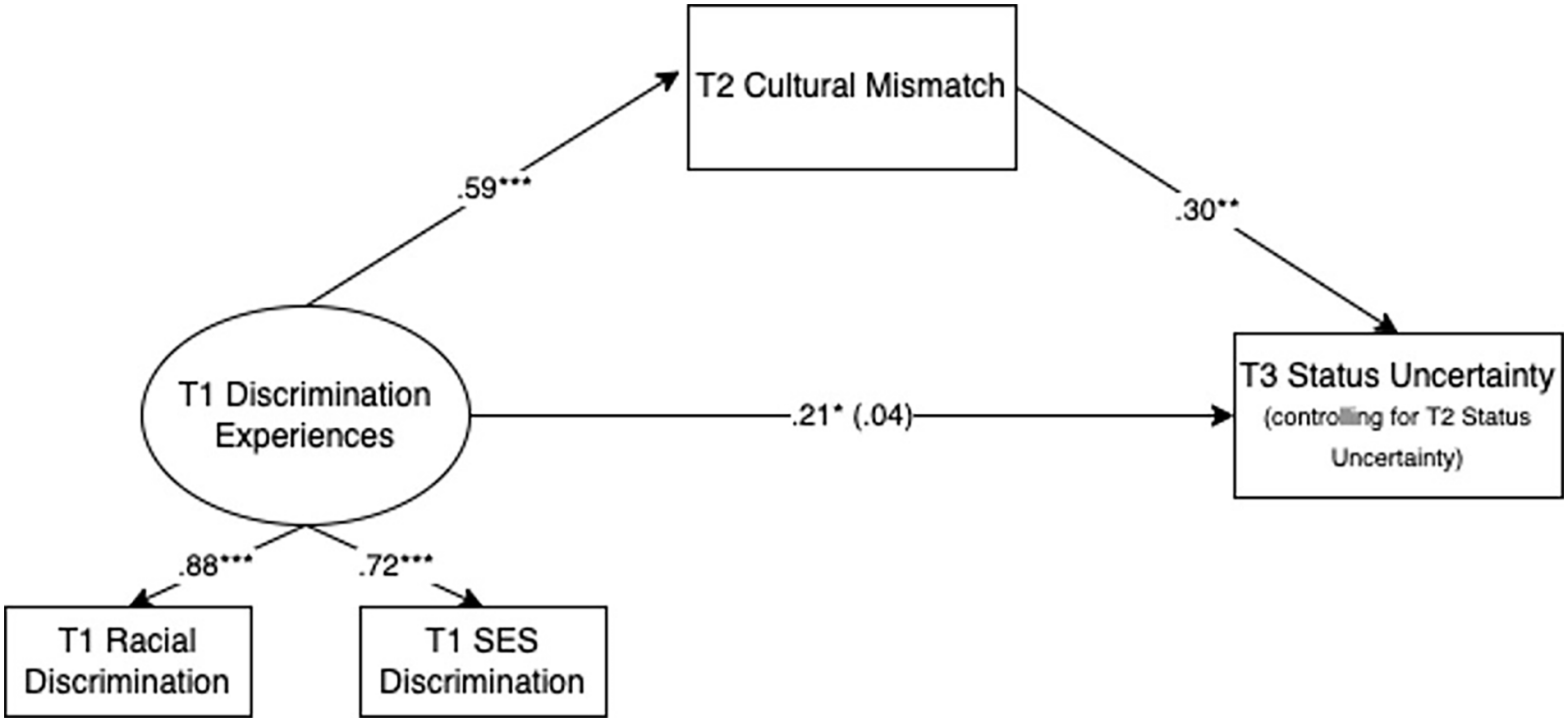
Model Results With Standardized Path Estimates and Factor Loadings. *p < .05. **p < .01. ***p < .001.

Model results are displayed in Table 2 and Figure 2. The total effect of discrimination experiences on status uncertainty was significant, β = .21, *p* = .016. As predicted, participants who experienced more racial and/or SES discrimination during their first year of college reported increased status uncertainty over their third year, controlling for status uncertainty at the end of their second year. Also as predicted, this effect was significantly mediated by perceived cultural mismatch within the university context. More frequent discrimination experiences at T1 significantly predicted greater perceived mismatch with the university environment at T2, β = .59, *p* < .001. In turn, greater perceived cultural mismatch at T2 significantly predicted increases in feelings of status uncertainty from the second to third years of college, β = .30, *p* = .003. The indirect effect of discrimination experiences on status uncertainty via cultural mismatch was significant, β = .18, *p* = .005. Furthermore, when accounting for perceived cultural mismatch, the direct effect of discrimination experiences on status uncertainty was no longer significant, β = .04, *p* = .763. This provides support for the proposed model that more frequent discrimination experiences predict greater feelings of cultural mismatch, which predicts increased status uncertainty over time. Results were unchanged when accounting for discrimination experiences at college entry and when tested among only low-SES students (and not first-generation college students) in the sample (see Supplemental Material).

**Table 2.** Results for Mediational Model.

Path	*B* (*SE*)	95% CI	*p*	β
Total effect	0.35 (.16)	[0.07, 0.68]	.016	.21
Indirect effect	0.29 (.10)	[0.11, 0.53]	.005	.18
Direct effect	0.06 (.20)	[−0.30, 0.49]	.763	.04
Path A	1.04 (.16)	[0.71, 1.35]	<.001	.59
Path B	0.28 (.10)	[0.08, 0.46]	.003	.30
Overall model fit	χ^2^(10) = 20.167, *p* = .028	RMSEA = .065, 90% CI [.021, .107]	CFI = .963	SRMR = .061

*Note.* CI = confidence interval; RMSEA = root mean square error of approximation; CFI = comparative fit index; SRMR = standardized root mean square residual.

### Alternative Models

In addition to the model presented in the main results section, we tested alternative models to examine which model seems to best explain the variability in status uncertainty observed in our data.

#### Testing University Fit Latent Variable as a Mediator

**Figure 3.**
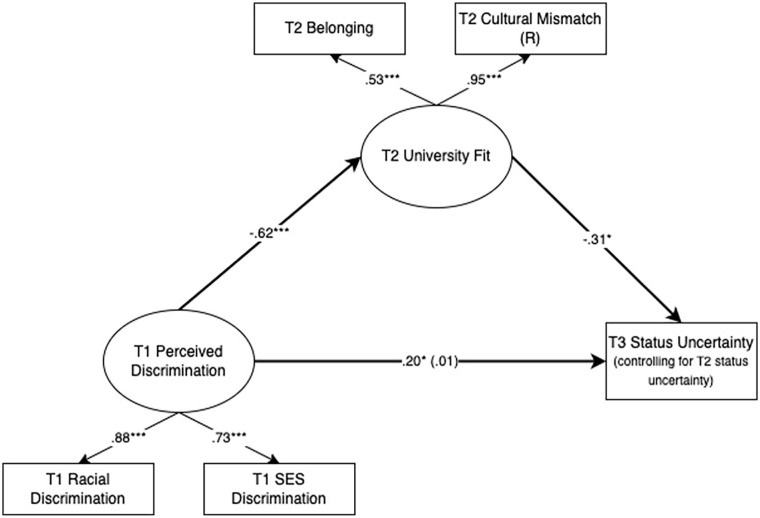
Model Results With Standardized Path Estimates and Factor Loadings Using University Fit Latent Variable as the Mediator. *p < .05. ***p < .001.

We tested an alternative mediation model that conceptualized university fit as the mediator of the effect of discrimination on status uncertainty (see Table 3 and Figure 3). University fit was modeled as a latent variable with two indicators: belonging and cultural mismatch (reverse-scored). These two measures were significantly correlated at T2 (*r* = −.51, *p* < .001). Belonging was measured at T2 using a 5-item scale adapted from Good et al. (2012). Participants indicated how much they felt they belonged at UCSB (e.g., “*I feel that I belong at UCSB*” and “*I feel like I am part of the UCSB community*”*)* on a scale of 1 (*Strongly Disagree*) to 7 (*Strongly Agree*). We averaged these items to form a composite at T2 (α = .87, *M* = 5.48, *SD* = 1.07). We controlled for objective SES, subjective SES, and T2 status uncertainty when predicting T3 status uncertainty. Results indicated that university fit significantly mediated the relationship between discrimination experiences and status uncertainty, β = .19, *p* = .034. More frequent discrimination experiences at T1 significantly predicted lower levels of belonging and greater perceived mismatch with the university environment at T2, β = −.62, *p* < .001. In turn, lower levels of university fit at T2 significantly predicted increased feelings of uncertainty around where one stands in society from T2 to T3, β = −.31, *p* = .012.

**Table 3.** Results for Model With University Fit Latent Variable as the Mediator.

Path	*B* (*SE*)	95% CI	*p*	β
Total effect	0.33 (.16)	[0.05, 0.67]	.040	.20
Indirect effect	0.31 (.15)	[0.09, 0.70]	.034	.19
Direct effect	0.02 (.23)	[−0.45, 0.48]	.950	.01
Path A	−0.51 (.13)	[−0.78, −0.26]	<.001	−.62
Path B	−0.62 (.25)	[−1.16, −0.18]	.012	−.31
Overall model fit	χ^2^(14) = 23.78, *p* = .049	RMSEA = .054, 90% CI [.004, .091]	CFI = .970	SRMR = .063

*Note.* CI = confidence interval; RMSEA = root mean square error of approximation; CFI = comparative fit index; SRMR = standardized root mean square residual.

#### Separating University Fit Latent Variable

To better understand whether these effects were being driven by cultural mismatch or by belonging, we separated the university fit latent variable into its two components and ran two separate models. The first model, which used only cultural mismatch as the mediator, was presented in the main results (see Table 2) and provided evidence that cultural mismatch significantly mediated the relationship between discrimination experiences and status uncertainty. The second model (see Table 4), which used only the variable of belonging as the mediator, revealed that belonging did not significantly predict status uncertainty, β = −.16, *p* = .050, and did not significantly mediate the relationship between discrimination and status uncertainty, β = .04, *p* = .098. These results of these two models demonstrate that the mediation of the university fit latent variable was driven by cultural mismatch, rather than belongingness. Based on this finding, the fact that the model with only cultural mismatch had better fit with the data than the model using the university fit latent variable, and the principle of parsimony which states that a simpler model is preferred (Raykov & Marcoulides, 1999), the model using only cultural mismatch as the mediator of the relationship between discrimination experiences and status uncertainty seems to best predict the variation in status uncertainty in the data.

**Table 4.** Results for Model Using Only Belonging as the Mediator.

Path	*B* (*SE*)	95% CI	*p*	β
Total effect	0.31 (.20)	[0.01, 0.76]	.054	20
Indirect effect	0.07 (.05)	[0.01, 0.20]	.098	.04
Direct effect	0.24 (.20)	[−0.05, 0.70]	.147	.15
Path A	−0.42 (.18)	[−0.76, −0.06]	.004	−.28
Path B	−0.17 (.08)	[−0.32, 0.01]	.050	−.16
Overall model fit	χ^2^(10) = 21.18, *p* = .020	RMSEA = .069, 90% CI [0.026, 0.110]	CFI = .946	SRMR = .061

*Note.* CI = confidence interval; RMSEA = root mean square error of approximation; CFI = comparative fit index; SRMR = standardized root mean square residual.

#### Separating Discrimination Experiences Latent Variable

Similar analyses were conducted by separating the discrimination experiences latent variable into its two components and running two separate models. In the first model (see Table 5), only the variable of racial/ethnic discrimination was used as the initial predictor of cultural mismatch, and, in turn, status uncertainty. Racial/ethnic discrimination significantly predicted cultural mismatch, β = .48, *p* < .001, which, in turn, significantly predicted status uncertainty, β = .32, *p* < .001. The indirect effect was also significant, β = .15, *p* = .001. The second model included only the variable of SES discrimination as the initial predictor (see Table 6). SES discrimination significantly predicted cultural mismatch, β = .40, *p* < .001, which, in turn, significantly predicted status uncertainty, β = .31, *p* < .001. The indirect effect was also significant, β = .12, *p* = .002. Thus, it seems that both racial/ethnic and SES discrimination are important in this process of predicting status uncertainty. Based on these results and theory that suggests both forms of discrimination are present and impactful among the population of interest (low-SES racial/ethnic minorities), the latent variable of discrimination experiences was maintained in the main model.

**Table 5.** Results for Model With Racial Discrimination as the Predictor.

Path	*B* (*SE*)	95% CI	*p*	β
Total effect	0.26 (.11)	[0.04, 0.48]	.021	.17
Indirect effect	0.23 (.07)	[0.12, 0.40]	.001	.15
Direct effect	0.03 (.12)	[−0.22, 0.28]	.812	.02
Path A	0.76 (.11)	[0.54, 0.96]	<.001	.48
Path B	0.31 (.08)	[0.15, 0.46]	<.001	.32

*Note.* CI = confidence interval.

**Table 6.** Results for Model With SES Discrimination as the Predictor.

Path	*B* (*SE*)	95% CI	*p*	β
Total effect	0.21 (.10)	[0.01, 0.41]	.034	.17
Indirect effect	0.15 (.05)	[0.07, 0.26]	.002	.12
Direct effect	0.06 (.11)	[−0.14, 0.28]	.576	.05
Path A	0.51 (.10)	[0.32, 0.68]	<.001	.40
Path B	0.30 (.08)	[0.13, 0.45]	<.001	.31

*Note.* SES = socioeconomic status; CI = confidence interval.

#### Examining the Directionality Between Variables

Because the relationships proposed in the current research (i.e., between discrimination and cultural mismatch, and between cultural mismatch and status uncertainty) have only been previously theorized and not yet empirically tested, we also conducted an analysis examining the directionality of these relationships. We used a cross-lagged panel model to test whether discrimination experiences predicted cultural mismatch, or vice versa, and similarly whether cultural mismatch predicted status uncertainty, or vice versa.

Discrimination experiences were measured at T1–T3, but cultural mismatch and status uncertainty were only measured at T2 and T3 in the current data. We conducted a cross-lagged panel analysis using all three variables (the discrimination experiences latent variable, cultural mismatch, and status uncertainty) at T2 and T3. Model fit was good, χ^2^(9) = 46.624, *p* < .001, root mean square error of approximation (RMSEA) = .138, 90% confidence interval (CI) = [0.100, 0.178], comparative fit index (CFI) = .949, standardized root mean square residual (SRMR) = .026. Results were not able to provide clarity on the directionality between discrimination experiences and cultural mismatch, as neither the effect of T2 discrimination predicting T3 cultural mismatch, β = .08, *p* = .370, nor the effect of T2 cultural mismatch predicting T3 discrimination, β = −.10, *p* = .304, were significant. The relationship between cultural mismatch and status uncertainty seems to be bidirectional as there was a significant effect of T2 cultural mismatch on T3 status uncertainty, β = .29, *p* < .001, as well as a significant effect of T2 status uncertainty on T3 cultural mismatch, β = .26, *p* < .001. Previous work on status uncertainty found that status uncertainty was cross-sectionally correlated with belonging and discussed belonging as a potential outcome, rather than predictor, of status uncertainty (Destin et al., 2017). Further research is needed to examine fit variables, such as perceived cultural mismatch and belonging, as both predictors and outcomes of status uncertainty.

## Discussion

During periods of upward mobility, people’s subjective experience of their status, or status-based identity, can be threatened, which can lead to status uncertainty. Emerging research indicates that status uncertainty is negatively associated with well-being, academic efficacy, and academic achievement. The current research contributes to this literature by examining predictors of status uncertainty. Specifically, we examined experiences during a time of social mobility—attending college—that can lead to increases in status uncertainty. Focusing on a sample of Latinx students who were either low-SES and/or first-generation college students, we proposed that experiencing discrimination in college and perceived cultural mismatch with college are key components of the process leading to feelings of status uncertainty. Specifically, we hypothesized that frequent discrimination experiences would predict greater feelings of cultural mismatch, which, in turn, would lead to increased feelings of status uncertainty.

As hypothesized, students who experienced more frequent discrimination during their first year of college based on their race/ethnicity or SES felt more mismatch between their background and the university culture by the end of their second year of college. To our knowledge, this is the first study to prospectively demonstrate a relationship between discrimination and perceptions of cultural mismatch. This finding is consistent with the idea that discrimination is a threat to the social identity of low-SES racial/ethnic minority students, which leads to feelings of incompatibility between their culture and background and the university context.

Also as hypothesized, greater perceptions of cultural mismatch with the university context predicted increases in status uncertainty from the end of students’ second year of college to the end of their third year. Although cultural mismatch had been theorized to be a predictor of status uncertainty (Destin et al., 2017), to our knowledge, this is the first study to demonstrate this empirically. Notably, the indirect pathway from discrimination experiences to status uncertainty via cultural mismatch was also significant. Students who experienced more frequent discrimination during the first year of college felt greater feelings of cultural mismatch 1 year later, which, in turn, led them to experience greater status uncertainty over the following year. Thus, this study also contributes to the literature by illuminating the process by which experiences during periods of social mobility, specifically discrimination and cultural mismatch, may lead to status uncertainty.

### Limitations and Future Directions

The current research focused on low-income and/or first-generation Latinx college students matriculating at a 4-year university where their ethnic group was in the minority. This sample was selected because these students are at a higher risk of experiencing discrimination based upon their race/ethnicity and their status and of feeling cultural mismatch with the university. However, our decision to focus on this population precluded us from having sufficient power to separately examine the impact of status versus race-based discrimination on status uncertainty and from examining the predictors of status uncertainty in students more advantaged on the dimension of ethnicity, SES, or both (i.e., lower income White students, higher income Latinx students, or higher income White students, respectively). We were also unable to test this process among different racial/ethnic minority groups, such as low-SES Black college students. We expect the results of the current study to generalize to populations with one or more disadvantaged identities because of the potential for these groups to experience discrimination on the basis of these identities, and, in turn, cultural mismatch within the university environment. However, for advantaged students at lower risk of experiencing discrimination and cultural mismatch, we suspect there may be a different set of variables important in shaping their experiences of status uncertainty. Future research might investigate what leads to status uncertainty in different contexts and in different populations.

The current study also focused on the transition from the end of students’ second year of college to the end of their third year. It would be helpful for future research to expand the timeframe in which status uncertainty is examined and test whether certain variables are stronger predictors of status uncertainty at specific timepoints during status transitions, such as when students first enter college or when they graduate. Expanding the timeframe when these variables are measured would also allow greater understanding of the directionality of these relationships. Future research can use causal modeling to incorporate changes in both discrimination experiences and cultural mismatch to examine associations with changes in status uncertainty.

The present study identified discrimination experiences and perceived cultural mismatch as predictors of status uncertainty but did not incorporate the consequences of status uncertainty in the current model. Prior research has found that status uncertainty, cultural mismatch, and discrimination all have negative impacts on well-being and academic achievement (Booker, 2004; Castillo-Lavergne & Destin, 2019; DeGarmo & Martinez, 2006; Destin et al., 2019; Schmitt et al., 2014). The current research examined status uncertainty as the final outcome, but future research might examine whether status uncertainty mediates the negative effects of discrimination and cultural mismatch on well-being and academic achievement.

Finally, future research might examine individual factors or interventions that may protect against experiencing status uncertainty. One potential factor is bicultural identity integration (BII), or bicultural individual’s perceptions of the intersection between their two cultures (Benet-Martínez & Haritatos, 2005). Research on social class BII suggests that first-generation students vary in their ability to integrate between the cultures associated with their working class background and the upper/middle-class university environment (Herrmann & Varnum, 2018a, 2018b). Students higher in social class BII who are better able to blend these two environments and reduce conflict between these two cultures may also feel more certainty around their standing in society. In addition, manipulating different paths of the current model may lead to interventions to protect against status uncertainty. Values affirmation interventions have been shown to reduce cultural mismatch among first-generation college students (Hecht et al., 2021). Similar interventions may be useful in reducing status uncertainty.

## Conclusion

Status uncertainty has negative implications for the well-being and academic outcomes of low-SES racial and ethnic minority students, but little is known about what experiences lead to status uncertainty. The current research investigated predictors of status uncertainty during college, and highlights the process by which discrimination experiences and cultural mismatch together predict increased status-based identity uncertainty. Research on social mobility often examines objective and subjective markers of status change, such as educational attainment or the perception that one has gained a higher social standing. However, to better understand why racial and ethnic minorities have diminished returns on social mobility compared with White individuals, future research must examine SES from a more holistic perspective with the goal of understanding how individuals experience and make sense of their own changing status.

## Supplemental Material

sj-docx-1-psp-10.1177_01461672231163736 – Supplemental material for Discrimination and Perceived Cultural Mismatch Increase Status-Based Identity Uncertainty Supplemental material, sj-docx-1-psp-10.1177_01461672231163736 for Discrimination and Perceived Cultural Mismatch Increase Status-Based Identity Uncertainty by Sierra H. Feasel, Tessa L. Dover, Payton A. Small and Brenda Major in Personality and Social Psychology Bulletin

## Notes

1. $50,000 was a threshold used by the University registrar to identify low-income families and determine financial aid need.
