# Supplementary material for: Discrimination and Perceived Cultural Mismatch Increase Status-Based Identity Uncertainty
Source: Pers Soc Psychol Bull. 2023 Apr 7;50(8):1251–62. doi: 10.1177/01461672231163736 (PMC11193322; doi:10.1177/01461672231163736)
Supplement: sj-docx-1-psp-10.1177_01461672231163736 – Supplemental material for Discrimination and Perceived Cultural Mismatch Increase Status-Based Identity Uncertainty [file sj-docx-1-psp-10.1177_01461672231163736.docx]

**Additional Sample Characteristics**

**Table S1.** Additional sample characteristics.

| Racial and Ethnic Identification | 245  1  21  5  3  3 | (89.4%) solely Latinx  (0.4%) also Black/African American  (7.6%) also White/Caucasian  (1.8%) also Asian/Pacific Islander  (1.1%) also Native American  (1.1%) also ‘Other’ (reported Iranian, Middle Eastern, & Portuguese) | | |
| --- | --- | --- | --- | --- |
|  |  |  | | |
| Nativity | 23  34  180  38 | (8.4%) not reported  (12.4%) born outside of U.S.  (65.7%) at least 1 parent born outside of U.S.  (13.9%) both parents born in U.S. | | |
|  | | | | |
| Family Income | 30  63  107  47  14  14 | (10.9%) not reported  (23.0%) less than $25,000  (39.1%) $25,000 to $49,000  (17.2%) $50,000 to $74,000  (5.1%) $75,000 to $99,000  (5.1%) $100,000 or more | | |
|  |  |  | | |
| First Generation Student Status | 11  168  96 | (4.0%) not reported  (61.3%) first-generation college student  (35.0%) continuing-generation college student | | |
|  | | | | |
| Parental Education | Mother’s Education: | | Father’s Education: | |
|  | 15  131  69  41  14  25 | (5.5%) unknown or not reported  (75.3%) not HS graduate  (25.2%) HS graduate, no college  (15.0%) HS graduate, some college  (5.1%) college graduate, no grad training  (1.8%) grad training | 28  135  65  32  12  3 | (10.2%) unknown or not reported  (49.3%) not HS graduate  (23.7%) HS graduate, no college  (11.7%) HS graduate, some college  (4.4%) college graduate, no grad training  (1.1%) grad training |
|  | | | | |
| First language | 3  105  5  162 | (1.1%) not reported  (38.3%) English  (1.8%) both English and Spanish  (59.1%) Spanish | | |

**Supplemental Analyses**

All alternative models below were tested using *Mplus* version 8.0 (Muthén & Muthén, 1998-2017). T0 objective and subjective status were included as covariates when predicting T3 status uncertainty. We also controlled for T2 status uncertainty when predicting T3 status uncertainty to model change over time.

**Testing Discrimination Experiences as the Mediator**

We tested a model using discrimination experiences as the mediator and cultural mismatch as the predictor. Because cultural mismatch was only measured at T2 and T3, one pathway of the model had to be cross-sectional. The first model included path a as cross-sectional with both cultural mismatch and discrimination experiences measured at T2 (see Table S2). However, there was no significant mediation and the indirect effect of cultural mismatch on status uncertainty through discrimination was not significant, $\beta$ = .03, *p* = .636. The second model included path b as cross-sectional with both discrimination experiences and status uncertainty measured at T3 (see Table S3). Again, there was no significant mediation, $\beta$ = .05, *p* = .397. Discrimination experiences do not mediate the effect of cultural mismatch on status uncertainty. This provides support for the directionality of the model proposed in the main paper with cultural mismatch as the mediator of the relationship between discrimination and status uncertainty.

**Table S2**. Results for model with discrimination experiences as the mediator, path a modeled cross-sectionally.

| Path | B (SE) | 95% CI | *p* | $\beta$ |
| --- | --- | --- | --- | --- |
| Total Effect | .30 (.07) | [.16, .43] | <.001 | .32 |
| Indirect Effect | .03 (.07) | [-.09, .17] | .636 | .03 |
| Direct Effect | .27 (.10) | [.06, .45] | .006 | .28 |
| Path A | .32 (.04) | [.24, .39] | <.001 | .61 |
| Path B | .10 (.22) | [-.27, .58] | .630 | .06 |
| Overall model fit | $\chi^{2}$(11) = 22.460, p = .021 | RMSEA = .070, 90% CI (.026, .112) | CFI = .957 | SRMR = .073 |

**Table S3**. Results for model with discrimination experiences as the mediator, path b modeled cross-sectionally.

| Path | B (SE) | 95% CI | *p* | $\beta$ |
| --- | --- | --- | --- | --- |
| Total Effect | .30 (.07) | [.16, .43] | <.001 | .32 |
| Indirect Effect | .04 (.05) | [-.05, .15] | .397 | .05 |
| Direct Effect | .25 (.09) | [.08, .42] | .003 | .27 |
| Path A | .31 (.05) | [.22, .41] | <.001 | .54 |
| Path B | .14 (.16) | [-.17, .47] | .382 | .08 |
| Overall model fit | $\chi^{2}$(11) = 15.269, p = .171 | RMSEA = .043, 90% CI (.000, .090) | CFI = .981 | SRMR = .068 |

**Testing a Model with No Mediation**

As another alternative model, we examined discrimination experiences and cultural mismatch as simultaneous independent predictors of status uncertainty, with no mediation. This model did not fit the data well, $\chi^{2}$(8) = 66.854, p < .001 , RMSEA = .194, 90% CI (.153, .239), CFI = .735, SRMR = .172. Results indicated that status uncertainty over time was only predicted by cultural mismatch, B = .28, SE = .09, 95% CI [.13, .47], $\beta$ = .29, *p* = .001, and not by discrimination, B = .15, SE = .26, 95% CI [-.45, .52], $\beta$ = .06, *p* = .616. This provides further support for the model presented in the main paper.

**Accounting for Discrimination Experiences at College Entry (T0)**

We tested an alternative model that accounted for discrimination experiences at college entry (see Table S4). Results did not change from the main paper findings. Discrimination experiences was a significant predictor of cultural mismatch, $\beta$ = .59, *p* < .001, which in turn, predicted increased status uncertainty over time, $\beta$ = .26, *p* = .008. The indirect effect was significant indicating significant mediation,$\beta$ = .16, *p* = .010.

**Table S4**. Results for model accounting for T0 discrimination experiences.

| Path | B (SE) | 95% CI | *p* | $\beta$ |
| --- | --- | --- | --- | --- |
| Total Effect | .44 (.15) | [.17, .76] | .002 | .26 |
| Indirect Effect | .27 (.10) | [.08, .49] | .010 | .16 |
| Direct Effect | .17 (.19) | [-.19, .58] | .352 | .10 |
| Path A | 1.09 (.13) | [.84, 1.34] | <.001 | .59 |
| Path B | .25 (.10) | [.06, .43] | .008 | .26 |
| Effect of T0 Disc on T1 Disc | .67 (.07) | [.54, .81] | <.001 | .81 |
| Overall model fit | $\chi^{2}$(22) = 44.013, p = .004 | RMSEA = .063, 90% CI (.035, .090) | CFI = .959 | SRMR = .067 |

**Testing Model Among Only Low-SES Students**

Because only about 75% of our sample was low-SES and the other 25% were first-generation college students (but not low-SES), we tested the proposed model among only low-SES students (N = 160) to ensure that results did not change (see Table S5). Indeed, results mirrored findings in the main paper when using only low-SES racial/ethnic minority students from our sample. Discrimination experiences was a significant predictor of cultural mismatch, $\beta$ = .59, *p* < .001, which in turn, predicted increased status uncertainty over time, $\beta$ = .33, *p* = .011. The indirect effect was significant indicating significant mediation, $\beta$ = .20, *p* = .012. However, there was no total effect of discrimination experiences on status uncertainty among this subset of the sample, $\beta$ = .21, *p* = .078.

**Table S5**. Results among only low-SES students.

| Path | B (SE) | 95% CI | *p* | $\beta$ |
| --- | --- | --- | --- | --- |
| Total Effect | .33 (.21) | [-.02, .80] | .078 | .21 |
| Indirect Effect | .32 (.13) | [.11, .60] | .012 | .20 |
| Direct Effect | .02 (.26) | [-.36, .64] | .951 | .01 |
| Path A | 1.03 (.20) | [.59, 1.40] | <.001 | .59 |
| Path B | .31 (.13) | [.05, .54] | .011 | .33 |
| Overall model fit | $\chi^{2}$(10) = 13.736, p = .185 | RMSEA = .048, 90% CI (.000, .105) | CFI = .979 | SRMR = .048 |

**Partial Correlations Among Study Variables Controlling for Self-Concept Clarity**

The status uncertainty scale developed by Destin and colleagues (2017) was based on the self-concept clarity scale and adapted the self-concept clarity items to be about social status. In the current data, self-concept clarity was only measured at T3, so we were unable to properly control for it in the model. However, we did look at cross-sectional partial correlations at T3 between status uncertainty and the model variables, racial discrimination, SES discrimination, and cultural mismatch, controlling for self-concept clarity. After controlling for self-concept clarity, status uncertainty was significantly positively correlated with SES discrimination, *r* = .18, *p* = .016, and cultural mismatch, *r* = .22, *p* = .003, but not with racial discrimination, *r* = .14, *p* = .063.

**Methodology File**

Materials and data can be accessed through the Open Science Framework: <https://osf.io/25anw/?view_only=46b37c2a9799495ca66b4b51573ff59e>
